# The Effect of Solution-Focused Group Counseling Intervention on College Students’ Internet Addiction: A Pilot Study

**DOI:** 10.3390/ijerph17072519

**Published:** 2020-04-07

**Authors:** Xinhe Zhang, Xiaoxuan Shi, Shuowei Xu, Jingwen Qiu, Ofir Turel, Qinghua He

**Affiliations:** 1 Faculty of Psychology, Ministry of Education Key Laboratory of Cognition and Personality, Southwest University, Chongqing 400715, China; guhong15@126.com (X.Z.); shixx@email.swu.edu.cn (X.S.); adson2688@gmail.com (S.X.); qjw990130@email.swu.edu.cn (J.Q.); 2 Information Systems and Decision Sciences, California State University, Fullerton, CA 92831, USA; oturel@fullerton.edu; 3 Chongqing Collaborative Innovation Center for Brain Science, Chongqing 400715, China; 4 Southwest University Branch, Collaborative Innovation Center of Assessment toward Basic Education Quality at Beijing Normal University, Chongqing 400715, China; 5 Institute of Psychology, Key Laboratory of Mental Health, Chinese Academy of Sciences, Beijing 100101, China

**Keywords:** Internet addiction, solution-focused group counseling, intervention, college students

## Abstract

This pilot study aimed to explore the effect of solution-focused group counseling intervention on Internet addiction among college students. Eighteen college students participated in this study, out of which nine subjects were assigned into the experimental group and the rest (*n* = 9) to a control group. The experimental group received group counseling for five weeks, while the control group did not receive any intervention. The revised version of the Chinese Internet Addiction Scale (CIAS-R) was used to capture pre-test and post-test excessive use in the two groups. The experimental group was also subjected to a follow-up test and self-reported Internet addiction scores six months after the end of group counseling. Results showed that after the five-week solution-focused group counseling, the scores of four dimensions of the CIAS-R in the experimental group had CIAS-R decreased, and the reduction trend of the total score of CIAS-R was similar across all subjects in this group. The treatment effect was larger than the placebo reduction in the control group in two dimensions: compulsive and withdrawal (Sym-C & Sym-W) and tolerance (Sym-T) symptoms. Qualitative research confirmed the conclusions from the quantitative data, showing that the experimental group reduced its Internet addiction symptoms. Overall, the findings suggested that solution-focused group counseling had positive intervention effects on Internet addiction.

## 1. Introduction

The Internet can serve as a double-edged sword. On the one hand, it provides many benefits and increases convenience. On the other, its use can be highly alluring, which may result in excess consumption, which manifests in behavioral addiction-like symptoms [1]. Since Goldberg [2] proposed the concept of “Internet Addiction Disorder”, many researchers have used similar concepts, such as Pathological Internet Use, Problematic Internet Use, Excessive Internet Use, and Internet Addiction [3].

Although these concepts are slightly different, they all have two common characteristics: (1) addiction-like symptoms: compulsive use of Internet, withdrawal reactions, and tolerance, combined with inability of users to effectively control themselves; (2) as a result, their daily functions such as academic and social activities, as well as sleep and food intake, are impaired [4]. We believe that the concept of Internet Addiction (IA) can best reflect the above characteristics and is widely used. While the term is controversial [5], for convenience reasons, we will use in this study the term Internet Addiction to describe this phenomenon, while acknowledging the need of future research to fine tune its definition and boundaries. IA has been recognized as a public health concern [6], which can correlate with poor health outcomes such as higher likelihood of being overweight [7], impaired sleep [8], increased suicidal ideation and attempt [9], and increased depressive symptoms [10]. 

College students can be highly susceptible to IA [11]. Once freed from the strict control of high school and family, they tend to have free time at their disposal, which when coupled with easy access to the Internet can lead to IA [12]. In addition, these students are experiencing changes in the environment with which they must learn to cope. For example, they face pressures on handling new interpersonal relationships and balancing learning and social activities [13]. Consequently, students may seek to relieve these pressures via Internet use, which can result in IA [14]. Among Chinese college students, IA is prevalent. Between 6.56% and 13.5% of Chinese college students may be classified as addicted to the Internet [15]. Therefore, paying close attention to issues related to college students’ use of the Internet, and intervening if necessary, is critical for preventing IA and its adverse impacts [16].

Various schools of psychotherapy have been applied to alleviate IA. Among them, solution-focused brief therapy (SFBT), as a relatively new paradigm of post-modern constructivism, has also been applied to IA cases. Specifically, Yang [17] provided psychotherapy with an emphasis on SFBT and comprehensive family intervention to teenagers with IA, and reported positive results showing that SFBT can be an effective therapy for treating IA. SFBT evolved out of the clinical practice of Steve de Shazer, Insoo Kim Berg, and colleagues at the Brief Family Therapy Center in Milwaukee, Wisconsin, in the early 1980s [18]. SFBT change processes were originally grounded in the constructivist approaches to communication and social interactional theories [19]. Over time, SFBT also became associated with social constructionism and the philosophical, post-structural views of language such as in Wittgenstein’s language games [20,21]. The main components of SFBT include looking for previous solutions, acknowledging problems but identifying that exceptions to the problem are key to the solution, focusing on present and future, as opposed to past-orientated questions, using validation, and utilizing techniques such as miracle questions, scaling questions, and coping questions [22]. 

Due to the high cost of psychotherapy, people strive for a faster and more effective treatment, which has led to the widespread application of SFBT, such as for treating students’ emotional behavior problems [23], substance abuse [24], adolescent crisis events, suicide and self-harm [25]. SFBT is not only applied to psychotherapy and counseling, but also applied in school class management, enterprise management and other fields [26]. SFBT emphasizes using a positive perspective to comprehend people, expressing their affirmation, and fully trusting that people will understand the methods that suit them. It is complementary to traditional Confucian culture [27] and compatible with it [28]. As such, it is highly suitable for application in China, as demonstrated by large effects in Chinese populations [29].

Comparing SFBT with Cognitive Behavioral Therapy (CBT), Jordan, Froerar, & Brovelas [30] argue that professionals using CBT assume the expert role, aiming to identify problems in people’s thinking and behavior. But professionals using SFBT regard their patients as already having all the resources they need, defining their own role as supportive in eliciting the individuals’ strengths, and empowering people to articulate these into achievable goals for the future. Therefore, SFBT enables patients to look for possible behaviors to change, and puts patients in control as regulators of their behavioral changes. It is not only beneficial for patients to maintain behavioral change, but also helps them to improve self-efficacy, thus making it more suitable for college students’ self-exploration and continuous transformation. In addition, some studies have shown that SFBT used more of the client’s exact words, and used more positive language, than other therapies such as CBT and Motivational Interviewing (MI) [30,31]. Therefore, SFBT may be more beneficial to relieve patients’ anxiety and their concerns towards the issue, thereby making it easier for college students who are sensitive and care about others’ evaluation and social pressures to accept this method and reduce their resistance. In addition, group counseling is an effective intervention for IA [32]. Compared to solution-focused individual counseling, applying SFBT to group counseling makes the process lively, encourages group members to share their experiences and promotes psychological growth with mutual support and encouragement from the group. Nevertheless, the efficacy of SFBT in group counseling to reduce IA in Chinese populations is not fully established. We aim at ameliorating this gap in this pilot study. 

Specifically, we focused on applying SFBT in group counseling and explored the intervention effects of solution-focused group counseling on IA in college students. By examining immediate and long-term post-intervention effects, we aim at clarifying if solution-focused group counseling improves IA in college students. Based on the above mentioned findings of prior intervention studies applying SFBT in different contexts, we hypothesized that participants in the experimental group (i.e., those who will accept five-week solution-focused group counseling aimed at alleviating Internet Addiction core symptoms, such as compulsive use, withdrawal and tolerance, and Internet Addiction related problems such as interpersonal, health-related, and time management problems) will show significant changes in these aspects, beyond the placebo effects observed in a control group.

## 2. Materials and Methods

### 2.1. Study Design and Participants

Participants were screened from a sample of 27 college students who presented high IA symptomology, recruited through flyers on campus. Based on an initial interview and the Revised Chinese Internet Addition Scale (CIAS-R), we selected 18 subjects with scores ≥53. The other 9 had score <53 and were excluded. The final 18 participants were randomly assigned to experimental or control groups with inclusion criteria CIAS-R score ≥53, which represents moderate to high levels of IA. There were 9 in the experimental group, (male = 3, female = 6; age = 20.11 ± 1.45) and 9 in the control group (male = 3, female = 6; age = 20 ± 1.56). Non-parametric Mann-Whitney U tests showed that there was no significant difference in CIAS-R score between the two groups [Z = −0.71, *p* > 0.05]. We also used a self-reported Symptom Checklist-90 (SCL-90) questionnaire as an auxiliary selection tool, and the scores of each factor of the students in the two groups were less than 5, showing no symptoms of serious mental disorders. The study was given ethical approval by the Institutional Review Board of the Southwest University (No. 20180030).

### 2.2. Procedures

Closed semi-structured group counseling was conducted on the experimental group once a week for five weeks. Every counseling session lasted about two and a half hours. There were one leader and two assistants in the group. The leader is a certified psychotherapist, and the assistants are psychology graduate students trained for group counseling. Their roles followed basic procedures that were designed in advance. Participants were given free interaction space. The leader regulated and navigated the group process according to the needs of the members. The control group did not receive any intervention. Both groups completed surveys at t1 and t2 (t1 + 5 weeks). The intervention group also completed a t1 + 6 months survey. Instruments included in the survey are outlined in the next section. 

### 2.3. Instruments

#### 2.3.1. Revised Chinese Internet Addiction Scale (CIAS-R)

The scale [33,34] consists of 19 items, on a four-point Likert scale. Two subscales are Internet Addiction Core Symptoms (IA-Sym) and Internet Addiction Related Problems (IA-RP). Core symptoms include four dimensions: Compulsive and Withdrawal Symptoms of Internet addiction (Sym–C&Sym-W), Tolerance Symptoms of Internet Addiction (Sym-T), Interpersonal and Health-Related Problems of Internet Addiction (RP-IH), and Time Management Problems (RP-TM). The experimental group and the control group completed the scale before and after group counseling, and the experimental group also carried out follow-up measurement six months after group counseling. The higher the score, the greater the risk of IA is. CIAS-R is widely used in the measurement of IA. Several studies show that CIAS-R has good reliability, with Cronbach’s alpha ranging from 0.85 to 0.90 [34,35,36]. In this study, Cronbach’s alpha was 0.86, 0.89 and 0.83 in the three rounds of administration.

#### 2.3.2. Symptom Checklist-90 (SCL-90)

The Symptom Checklist-90 (SCL-90) has a total of 90 items rated on a five-point Likert scale. It includes 9 factors, such as depression and anxiety. Studies have found that IA may be comorbid with depression and anxiety disorders [37,38]. If comorbidity exists, it is very important to evaluate and treat related mental disorders. However, our group counseling mainly focused on Internet addiction and had no strong pertinence to other mental disorders. Therefore, SCL-90 is used to screen whether the subjects had major comorbid mental disorders.

#### 2.3.3. Scaling Questions Form

Scaling questions are representative questions of SFBT. Members of the experimental group are required to evaluate the current state of Internet use with a 10-point score, which increases successively from 0 (“the worst state of Internet use”) to 10 (“the most ideal state of Internet use”). The experimental group completed the evaluation at the pre- and post-group counseling.

#### 2.3.4. Satisfaction Survey

The experimental group was asked to evaluate the overall satisfaction with group counseling at the end of each session on a 10-point Likert scale (from 0 = “very unsatisfied” to 10 = “very satisfied”).

#### 2.3.5. Change Questionnaire after Group Counseling

Six months after group counseling, the experimental group members were tracked by a change questionnaire, to examine the sustainability of changes after receiving the group counseling. The questionnaire employed open-ended questions that asked whether there was any change in the behavior of members after they participated in group counseling compared with before, such as Internet use time, attitude towards Internet use, behaviors related to Internet use, and changes in other life aspects beyond Internet use.

### 2.4. Data Analysis 

Quantitative data were analyzed with SPSS 23.0 (IBM Corp. Released, Armonk, NY, USA). We used the non-parametric Mann-Whitney U test and Friedman test for analyses. Qualitative data were sorted based on the Kawakita Jiro (KJ) method which is used to organize data into useful categories. It adopts the bottom-up sorting process and is very useful for classifying data. Based on the statement of significance of “Change after group counseling”, researchers summarized, coded and recorded responses with concise, independent and clear phrases, extracted representative topics, and formed a comprehensive database of self-reported parsed statements from group members.

### 2.5. Solution-Focused Group Counseling Programs

The Solution Focused Brief Therapy Association listed three elements of SFBT [39]. First, there are the overall topics. SFBT conversations are centered on client concerns: who and what are important to the clients; a vision of a preferred future; clients’ exceptions, strengths, and resources related to that vision; scaling of clients’ motivational level and confidence in finding solutions; and ongoing scaling of clients’ progress toward reaching the preferred future. Second, SFBT conversations involve a therapeutic process of co-constructing altered or new meanings in clients. Third, therapists use a number of specific responding and questioning techniques that invite clients to construct a vision of a preferred future and draw on their past successes, strengths, and resources to make that vision a reality. Based on the above factors we designed five focused-solution group counseling sessions (Table 1), with the purpose of assisting college students to establish the goal of alleviating IA (e.g., Ask group members: what changes about IA do you want to make after our group counseling sessions?), reconstruct the problem (e.g., Let group members reflect on the pros and cons of the internet through the group counseling sessions, discussing and sharing with each other), expand positive resources and exceptional experience (e.g., Ask group members: in your past life, to some extent, did you ever think that your internet usage has reached the target state?), and actively seek and enrich solutions (e.g., Let the group members list obstacles they may encounter when using the internet in the future, proving better solutions, after brainstorms.). In group counseling, the leader encouraged and assisted group members to share their experiences (e.g., All members were invited to participate in the group counseling sessions, freely expressing their views and feelings.), and actively adopt a variety of questions (e.g., When group members share twice regarding their changes, the leader will ask more details about what caused these changes.), compliments (e.g., At the end of each session, praise is given to the group members on their efforts to solve and conquer the problems of IA.), other techniques in SFBT (e.g., miracle question: When you go back to sleep in the dormitory tonight, a miracle will happen and all the problems that you brought here have all been solved. (Pauses) But because you are sleeping, you don’t know that a miracle has happened. When you wake up the next day, what will you pay attention to, in order to know that a miracle has happened?).

## 3. Results

### 3.1. Effects of Group Counseling Programs

The change in scores between t1 and t2 was used to compare the treatment and the control group changes (△= post-test score − pre-test score). We compared the scores with the non-parametric Mann-Whitney U test. The changes in both groups are shown in Table 2. As can be seen, both groups showed reduction in IA symptoms, but the reductions in the experimental group were larger. Specifically, the intervention produced significantly larger reductions, compared to the control group, in two IA symptoms: compulsive use and withdrawal (Sym-C & Sym-W) and tolerance (Sym-T), as well as in the total IA score. The small reduction in the control group may manifest from learning and social desirability.

### 3.2. Long-term Effects of the Intervention

**Figure 1:**
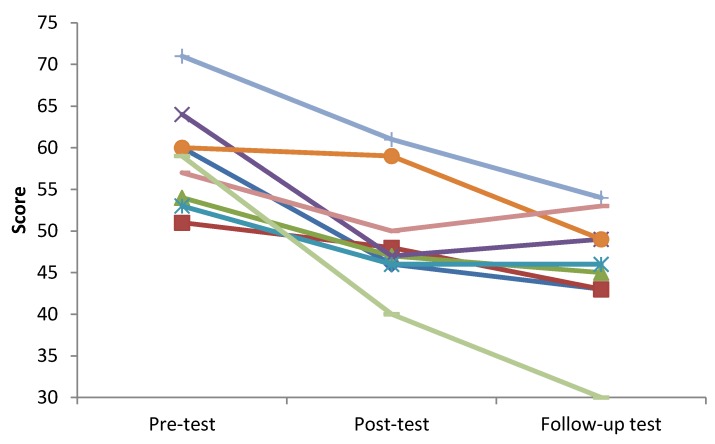
The Total IA Score in the Experimental Group (*n* = 9).

To examine long-term effects of the intervention, beyond the immediate post-intervention effects, we followed up with participants six months after t1. The results of the non-parametric Friedman test showed that significant effects existed in all dimensions of IA (Table 3). Statistical analysis of the data using the Wilcoxon signed-rank test revealed that the IA scores in the pre-test were significantly higher than those in the post-test and follow-up tests for three dimensions of IA: compulsive and withdrawal (Sym-C & Sym-W), tolerance (Sym-T) and time management problems (RP-TM), as well as for the total IA score. In the dimension of interpersonal and health-related problems (RP-IH), the scores of the pre- and post-test were significantly higher than those of the follow-up test. As shown in Figure 1, the change trend of the total score of the CIAS-R was similar for all subjects in the experimental group.

### 3.3. Survey Score in Group Counseling of Experimental Group

As shown in Table 4, the satisfaction degree of the experimental group for each group counseling was above 8.5. Paired-samples t-test revealed that the score of the experimental group on the scaling question at the last group counseling was significantly improved, compared with the first group counseling session (t = 8.00, *p* < 0.01).

### 3.4. The Change Questionnaire after Group Counseling of Experimental Group 

Based on the change questionnaire after group counseling, the changes of college students can be summarized as “changes related to Internet use” (i.e., changes are directly related to Internet use, including Internet use time, Internet use attitude, Internet use form, etc.) and “changes related to daily life” (i.e., changes in other aspects of daily life that are not directly related to Internet use, such as schedule, interpersonal communication, studying, etc.) (Table 5).

## 4. Discussion

This pilot study explored the intervention effect of solution-focused group counseling for college students’ IA. The results of the comparison between the two groups found that the change in total IA scores as well as in the dimensions of compulsive use, withdrawal (Sym-C & Sym-W) and tolerance (Sym-T) were significantly larger in the intervention compared to the control group. Within the experimental group, the scores in the four IA dimensions were significantly decreased and the change trend of the total IA score was similar for all subjects in the experimental group. In the dimension of time management problems (RP-TM), the follow-up test was significantly lower than the scores in the pre- and post-tests. In the other dimensions, the post-test was significantly lower than the pre-test, and the follow-up test was significantly lower than the pre-test. The score on the scaling question in the post-test in the experimental group was significantly higher than that in the pre-test, indicating that the members of the group subjectively felt improvements in IA after the group counseling. In addition, the open-ended responses revealed that Internet use time, attitudes toward Internet use and Internet use form, as well as many aspects of daily life of group members, have changed. Moreover, the satisfaction score of the experimental group for every-week group counseling was above 8.5 points, which showed that members valued the provided group counseling. Overall, the results described above indicated that solution-focused group counseling intervention has a positive effect on college students’ IA and can lead to healthier Internet use and lifestyles.

After group counseling, the experimental group experienced more significant changes than the control group in IA-Sym, including but not limited to compulsion and withdrawal symptoms (Sym-C & Sym-W), and tolerance symptoms (Sym-T). However, there were no significant differences between the experimental group and the control group with regards to interpersonal and health-related problems (RP-IH), time management problems (RP-TM), and other related IA issues. These results indicated that the characteristics and advantages of SFBT make this therapeutic approach highly targeted, symptom specific, and efficient. Specifically, SFBT helps group members to clarify their goals and find clues to realize their goals by helping them visualize themselves after they use the Internet in a healthier and responsible fashion and avoid IA symptoms. SFBT is effective for several reasons. It guides group members to actively think about the function of the Internet, and to restructure their negative attitudes about the Internet towards more positive views of Internet use. SFBT encourages members to actively explore positive experiences and coping strategies for rational uses of the internet, and to search for past successful experiences to help discover and replicate effective methods of overcoming IA and uncontrolled use. All of this is done in combination with brainstorming, interpersonal communication and sharing, and other related processes to enrich the treatment milieu. SFBT stimulates group members to put various methods into practice through homework, thus gradually boosting changes in members’ core IA symptoms. It should be noted here that the change in the experimental group is more significant and may be related to the effect of social desirability. Specifically, in the process of participating in group counseling, the experimental group establishes relationships with leaders and other members, and become eager for recognition and praise. This, coupled with clear goals of group counseling, may stimulate members to actively perform in line with group goals. Of course, this is also one of the motivations of the group. From the perspective of SFBT, the socially desirable responding of the parties is an important psychological resource, which can be used for achieving better results. Through asking questions, the parties can express their abilities and efforts in the process of answering, so that the parties can have a clearer understanding of how to make progress.

Research findings within the groups showed that the experimental group experienced positive significant changes in IA. The classification we employed is based on a study of a sample of 388 college students who completed the CIAS-R revised. They found that a cutoff of 46 separates normal from at-risk groups, and a cutoff of 53 separates at-risk from the Internet addiction group [32]. According to this standard, the average Internet addiction score of the experimental group in this study belongs to the range of Internet addiction before intervention, the range of Internet addiction risk after intervention, and the normal range after six months. Although it is only a rough comparison of the average value, it reflects the clinical significance of the intervention effect to a certain extent. However, the experimental group showed different changes in different aspects of Internet addiction. In terms of core IA symptoms (IA-Sym), such as compulsion (Sym-C), withdrawal (Sym-W) and tolerance symptoms (Sym-T), and time management problems (RP-TM), the immediate effect of group counseling was quite positive and lasted to the follow-up test, six months later. In the Interpersonal and health-related problems (RP-IH) domain, there was no significant change after group counseling, but there was a significant improvement six months later. In addition, the open-ended reports after the follow-up period supported the quantitative data, indicating that experimental group members had experienced positive changes in Internet use and daily life. An earlier study [29] found that the application of SFBT in China can have positive immediate effect. Extending this view, the current study demonstrated that in the form of group counseling SFBT can have both short-term and long-term effects. Additionally, the solution-focused group counseling of this study first stimulated changes in core problems, and those changes were mostly maintained after group counseling. While changes in other dimensions were not as rapid and obvious as changes in core problems, there was still the possibility of change after the group counseling. De Jong and Berg [40] suggested that the professional value of SFBT is multidimensional. It encourages clients’ involvement, improves clients’ self-determination, maximizes their sense of empowerment, and promotes transferability. Specifically, SFBT helps clients to help themselves, maximizes client potential and resources in solving problems, and makes clients responsible for their own lives by applying what they got from the counseling to other scenarios through the process of creating solutions with counselors. Therefore, solution-focused group counseling not only achieved significant outcomes in treating core IA symptoms, but also had positive longer-lasting impacts on interpersonal relationships, learning, work and other life domains.

Many researchers have also conducted intervention studies on college students’ Internet addiction, but follow-up tests are often conducted within six or eight weeks after the intervention [34,41]. We extend such studies in this paper, by focusing on long-term follow-up changes. Moreover, Cognitive-Behavioral Therapy has been widely applied to treatment of IA [42], but the existing cognitive behavioral therapies mostly draw on results of well-developed studies in substance addiction and intervention treatments of other mental disorders. These treatments may not be as efficacious with college students, which is a specific clinical group that needs to use the Internet, but needs to learn to do so responsibly, even under conditions of total freedom. These reasons may account for the limited success of these therapies with IA cases [43]. Besides shedding factors that have controlled their life, many college students lack self-control and behavior-selection strategies. Even if students make positive changes, these can be difficult to maintain. Moreover, each relapse to an addictive behavior will further undermine their self-efficacy [44], thus leading to increased engagement in addictive behaviors and further loss of control. Based on the concept of SFBT, the current pilot study respected the subjectivity of college students and helped them dig deeper into their own resources and strengths to solve the problem. In addition, the form of group counseling promoted positive feedback among members. This is not only conducive to the improvement in the core IA symptoms, but also helps to maintain the change and to generalize the change to other life domains.

Several limitations of the current study are noteworthy. First, the sample size is small. The reliability and validity of major measurement tools such as CIAS-R cannot be fully verified, and the generalizability of the results may be limited. Future studies can consider increasing the sample size to verify the reliability and validity of the measurement tools and improve generalization of the research results. Second, the variables in this study are all self-reported, subjective and easy to be influenced by the effect of social desirability. Future research can consider obtaining more objective data from different approaches such as multi-subject evaluation by consultants, classmates, etc., and/or control for social desirability bias. Third, the sample included college students. The design of the focus solution group counseling program is based on college students’ cognitive development level and other psychological characteristics, and the Internet addiction level of the subjects is controlled. In the future, when the group counseling program is applied to other age, identity or Internet addiction level groups, it needs to be revised according to the specific situation. Fourth, changes brought by group counseling may be subtle and even unconscious. More sensitive indicators, such as using neurophysiology related technologies to explore brain structure and functions following SFBT [43], can be used in future research to lay a foundation for improving IA interventions. 

## 5. Conclusions

In sum, this pilot study used solution-focused group counseling to treat college students with moderate to high levels of IA symptoms. The results suggested that solution-focused group counseling had positive effects on IA and some of its core dimensions, and was generally positively valued by participants. SFBT has the characteristics of high-efficiency, simplicity and durability in intervening with college students’ Internet use. We call for further studies to apply this approach and examine its merits. 

## Figures and Tables

**Table 1:** Solution-Focused Group Counseling Programs.

Theme	Objective	Activity Process
You and I together	Preliminary understanding between group members; Clarify and establish a group contract to be observed by all members of the group; Arouse the interest of individuals in participating groups; Clarify members’ expectations of the group and define members’ personal goals.	Warm up: The wind blows Serial self-introduction Form a group contract Clarify members’ personal goals Complete the scaling question form Feedback and homework: try to improve your score on scaling questions by 1 point; Think about and document the positive aspects of the Internet Complete the satisfaction survey
When miracles happen	Reconstruct the problem of IA, discuss the change between group counseling interventions and a vision of a preferred future, and dig out the materials to solve the problem.	Members share their change Warm up: In the same boat Share homework, reconstruct the problem of IA Progressive muscle relaxation and miracle question Feedback and homework: Think about and document the positive aspects of the Internet use Complete the satisfaction survey
Look for a successful self	Explore exceptional experiences and successful experiences that have been achieved, expand positive resources, and gain confidence.	7. Members share changes based on homework 8. Warm up: Trip to trust 9. Explore the exceptional experiences associated with internet use by group members 10. Feedback and homework: Replicate the exceptional experience 11. Complete the satisfaction survey
Make friends with the future	Discuss potential obstacles in the future and find and enrich solutions.	Members share changes based on homework Warm up: Break through the dilemma Brainstorming: Discuss solutions to obstacles in the future Leader gives a keynote speech: The content aims at the common trouble of the members of the previous three sessions—procrastination Feedback and homework: Replicate the exceptional experience Complete the satisfaction survey
Common agreement	Deal with the parting mood, discuss and summarize the changes brought by the group counseling to the members, praise the members and end the group.	King and angel: assign roles Warm up: Copy the human body Share the overall changes from five sessions of group counseling The angel wanted to talk to the king: Members compliment each other End group counseling: Invite members to finish outcome questionnaire after six months Complete the satisfaction survey and scaling question form

**Table 2:** Comparison between the Experimental and Control Groups.

Measures	Experimental Group (*n* = 9)	Control Group (*n* = 9)	Z
Sym-C & Sym-W	−2.67 ± 2.17	−0.88 ± 1.05	−1.99 *
Sym-T	−2.78 ± 1.72	−0.78 ± 1.20	−2.48 *
RP-IH	−2.00 ± 2.83	−0.11 ± 2.15	−1.48
RP-TM	−2.00 ± 2.45	−0.89 ± 1.54	−0.82
Total IA	−9.44 ± 6.13	−2.67 ± 4.24	−2.26 *

Note: Sym-C: compulsive use; Sym-W: withdrawal; Sym-T: tolerance; RP-IH: interpersonal and health-related problems; RP-TM: time management problems; * *p* < 0.05.

**Table 3:** Comparison of the Differences among Pre-test, Post-test and Tracking Scores in the Experimental Group (x ± s).

Measures	Pre-Test (T1)	Post-Test (T2)	Follow-up Test (T3)	χ2	Multiple Comparisons
Sym-C & Sym-W	17.44 ± 1.67	14.78 ± 1.86	13.89 ± 2.67	11.46 **	T1 > T2, T1 > T3
Sym-T	12.78 ± 1.72	10.00 ± 1.94	9.89 ± 2.20	13.15 **	T1 > T2, T1 > T3
RP-IH	15.89 ± 2.09	13.89 ± 2.57	11.67 ± 2.78	13.24 **	T1 > T3, T2 > T3
RP-TM	12.67 ± 1.73	10.67 ± 2.29	10.33 ± 1.32	11.53 **	T1 > T2, T1 > T3
Total IA	58.78 ± 6.12	49.33 ± 6.63	45.78 ± 7.12	14.80 **	T1 > T2, T1 > T3

Note: Sym-C: compulsive use; Sym-W: withdrawal; Sym-T: tolerance; RP-IH: interpersonal and health-related problems; RP-TM: time management problems; ** *p* < 0.01.

**Table 4:** Satisfaction Survey Score of Experimental Group.

Measures	Week 1	Week 2	Week 3	Week 4	Week 5
Satisfaction Score	8.78 ± 0.63	8.75 ± 0.83	9.67 ± 0.47	9.33 ± 0.47	9.78 ± 0.42
scaling question Score	3.11 ± 0.57	/	/	/	5.78 ± 0.92

**Table 5:** Benign Changes after Group Counseling of Experimental Group Members.

Third-Level Coding	Second-Level Coding	First-Level Coding (Number of People Mentioned & Examples of Group Members’ Changes)
Changes related to the Internet use	Changes in Internet use time	Time spent on Internet reduced (4; e.g., Time reduced by about an hour or two)
		Reasonable planning and control of Internet use time (2; e.g., Able to put the phone down in time)
		Reasonably allocate time for online entertainment and learning (3; e.g., Study for about 40 minutes, and take a 10-minute break to play on the phone)
		Time spent on games reduced (1)
	Changes in Internet use attitude	Objective view on Internet use (4; e.g., Understand my own Internet use behavior)
		When surfing the Internet, my mentality is more peaceful (2; e.g., Not experiencing dilemmas like before on using internet)
		The appeal of my mobile phone declined (1)
		More focused on other things (1)
		It doesn’t matter if I don’t surf the Internet (2; e.g., It’s no big deal not to be online)
	Changes in Internet use form	The Internet becomes a relaxation tool (3 e.g., Occasionally watch videos to relax)
		The Internet becomes a learning tool (1)
		The Internet becomes a tool of daily life (2; e.g., Now usually use Internet to chat)
		More meaningful content browsed on the Internet (1)
		Dialectical view of Internet point (1)
Changes related to daily life	Changes in schedule	Schedule more regular (2; e.g., Meals on times)
		Internet use will not overly affect sleep (1)
		More days to get up early (1)
		The number and timing of getting up lately adjusted (1)
	Changes in work and study	Spent time on reflection (1)
		More time spent on study (1)
		The number of visits to the library increased (1)
	Changes in interpersonal communication	More activities with friends (2; e.g., Spend more time out with friends for studying and playing)
		Internet use will not affect interpersonal communication (1)
	Changes in life planning	Re-adjust life planning (1)
		Life plan is clearer (1)
	Changes in whole life	Use time to adjust life (1)
		Life is more organized (1)
	Changes in other aspects	The amount of exercise increased (1)
		Internet use will not affect walking (1)
		Began to pay attention to clothing collocation (1)
		Pay more attention to observing emotions (1)
		Continuous counseling (1)
